# A Novel Prokaryotic Promoter Identified in the Genome of Some Monopartite Begomoviruses

**DOI:** 10.1371/journal.pone.0070037

**Published:** 2013-07-25

**Authors:** Wei-Chen Wang, Yau-Heiu Hsu, Na-Sheng Lin, Chia-Ying Wu, Yi-Chin Lai, Chung-Chi Hu

**Affiliations:** 1 Graduate Institute of Biotechnology, National Chung Hsing University, Taichung, Taiwan; 2 Institute of Plant and Microbial Biology, Academia Sinica, Taipei, Taiwan; University of California, Riverside, United States of America

## Abstract

Geminiviruses are known to exhibit both prokaryotic and eukaryotic features in their genomes, with the ability to express their genes and even replicate in bacterial cells. We have demonstrated previously the existence of unit-length single-stranded circular DNAs of Ageratum yellow vein virus (AYVV, a species in the genus *Begomovirus*, family *Geminiviridae*) in *Escherichia coli* cells, which prompted our search for unknown prokaryotic functions in the begomovirus genomes. By using a promoter trapping strategy, we identified a novel prokaryotic promoter, designated AV3 promoter, in nts 762-831 of the AYVV genome. Activity assays revealed that the AV3 promoter is strong, unidirectional, and constitutive, with an endogenous downstream ribosome binding site and a translatable short open reading frame of eight amino acids. Sequence analyses suggested that the AV3 promoter might be a remnant of prokaryotic ancestors that could be related to certain promoters of bacteria from marine or freshwater environments. The discovery of the prokaryotic AV3 promoter provided further evidence for the prokaryotic origin in the evolutionary history of geminiviruses.

## Introduction

Geminiviruses are a unique family of plant viruses, consisting of four genera, *Mastrevirus*, *Curtovirus*, *Begomovirus*, and *Topocuvirus*, based on host ranges, vector specificities, and genome organizations [1,2]. Apart from the characteristic geminate-shaped virus particles and the single-stranded circular DNA genomes, geminiviruses have crossed the boundary of prokaryotes and eukaryotes [3,4]. Even within the eukaryotic host plants, genomic and various DNA forms possibly involved in the replication of geminiviruses have been identified in the plastids [5–8], the organelles of prokaryotic origin that shares many features with prokaryotes, including bacterial- or phage-type RNA polymerases for transcription (for a recent review, see 9), and structural similarities with prokaryotic ribosomes and nucleotide sequence homology with 16S rRNA [10] for translation. These unique features are indicative of the prokaryotic origin of geminiviruses [11–14].

Geminiviruses do not encode viral DNA polymerases and depend on host machineries for replication and transcription [15]. The replication-associated protein (Rep) of geminiviruses has exhibited similarities with those of prokaryotic plasmids [16]. The major replication mechanism of geminiviruses is through rolling-circle replication [17], which is analogous to prokaryotic systems found in a class of eubacterial plasmids and some bacteriophages [18]. Previous studies have demonstrated that some geminivirus species could express their genes and even replicate in bacterial cells [3,4]. Furthermore, we have shown previously that unit-length, single-stranded circular DNAs of both virion- and complementary-senses of Ageratum yellow vein virus (AYVV) could be generated in *Escherichia coli* (*E. coli*) cells harboring single copies of the AYVV genome [19]. These observations suggest that certain prokaryotic features of geminiviruses may be retained and still be functional in their infection cycles, including unknown functions yet to be elucidated.

In this study, we explored the AYVV genome further to search for the presence of additional prokaryotic promoter regions. A novel prokaryotic promoter, the AV3 promoter, was identified near the 3’-terminus of the coat protein (CP) gene, in which no promoters of known geminivirus genes have been reported. Further characterization of the AV3 promoter revealed that it is unidirectional, constitutive, and relatively strong, with the ability to drive the expression of a short open reading frame (ORF) in *E. coli*. The presence of the AV3 promoter in begomoviruses further solidifies the prokaryotic origins of geminiviruses.

## Materials and Methods

### Viruses

*Ageratum* *yellow* *vein virus* isolate NT (AYVV-NT) was originally isolated from infected *Ageratum* *conyzoides* from Nan-Tou County, Taiwan. The whole genome of AYVV-NT has been cloned into the *Bam*HI site of pUC119 to yield pAYVV-NT, and has been completely sequenced (GenBank accession EF458639 [19]). The isolates of *Gonostegia* *mosaic* *virus* (GoMV), Tomato leaf curl virus (TLCV), and *Squashleaf* *curl virus* (SqLCV) have been described previously [19,20].

### Promoter Trapping

**Figure 1:**
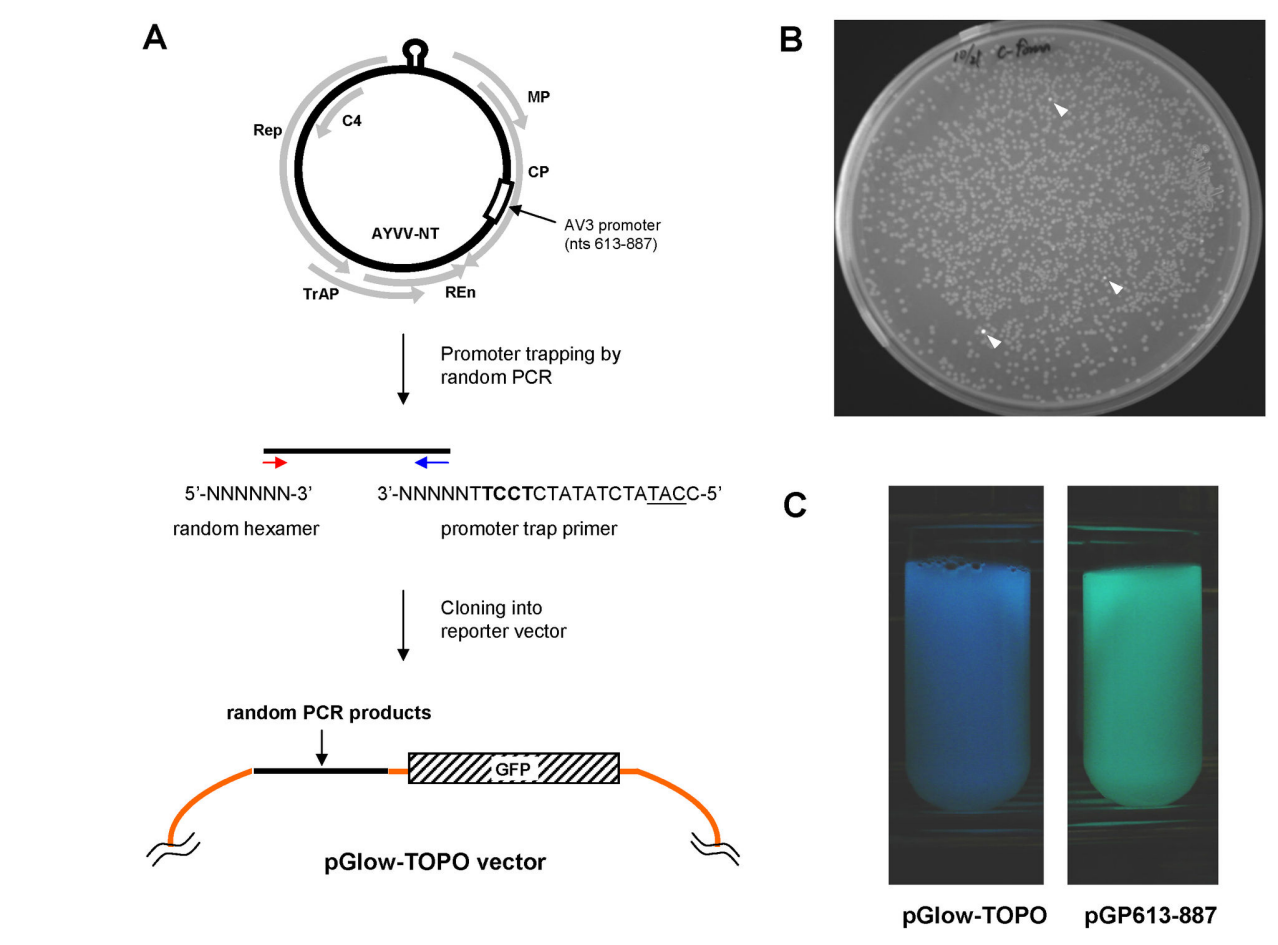
Identification of promoter activities in *E. coli*. (A) A schematic representation of the genomic map of AYVV-NT and the strategy used to randomly amplify fragments of AYVV-NT genome. The positions and directions of the known ORFs are indicated by the grey arrows. Random PCR products were cloned into the promoter trapping vector, pGlow-TOPO^®^ to identify the fragments with potential prokaryotic promoter activities in *E. coli*. The position of AV3 promoter is indicated by open box. (B) Examination of colonies with a handheld UV illuminator at 365 nm. The fluorescent colonies are indicated by the white arrowheads. (C) One selected colony (as indicated) was grown in the LB medium and examined under UV illumination.

To search for unknown prokaryotic promoters in the AYVV-NT genome, a promoter trapping strategy (Figure 1A) was adopted by using a TOPO^®^ Reporter Kit (Invitrogen, life technologies, Carlsbad, CA, USA) and following manufacturer instructions. The promoter trapping vector pGlow-TOPO is originally designed for promoter activity assays in mammalian systems, and therefore does not contain a prokaryotic ribosome binding site (RBS) upstream of the green fluorescence protein (GFP) ORF. To facilitate the efficient translation of the GFP reporter in *E. coli*, the RBS, start codon, and a spacer sequence (usually 8-12 nucleotides) in between must be artificially added to the promoter trap primer. Thus, the promoter trap primer, 5’-CC A TATCTATATC**TCCT**TNNNNN-3’, was designed to amplify random fragments of the whole AYVV-NT genome by polymerase chain reaction (PCR), when paired with random hexamer. The underlined nucleotides are the initiation codon in-frame with Cycle 3 GFP ORF on the pGlow-TOPO® vector, those in boldface are the prokaryotic RBS, whereas those in italics are the 9-nt spacer sequence identical to that used in the pET21d vector (Novagen, Billerica, MA, USA [21]) to provide proper spacing for efficient translation in *E. coli*. The 3’ end of the primer contains five random nucleotides (N) for base-pairing with AYVV-NT genomic sequences.

Amplification by using PCR was programmed to perform one cycle of 5 min at 94 °C, followed by 35 cycles of 30 s at 94 °C, 30 s at 37 °C, and 1 min at 72 °C, with a final extension step of 1 min at 72 °C, using the promoter trap primer and random hexamer as the primer pair and AYVV-NT genomic DNA as the template. The PCR products were cloned into the pGlow-TOPO^®^ vector by following manufacturer instructions, and were transformed into TOP10 *E. coli*. The plates were incubated at 37 °C for 16 h, and the colonies harboring putative prokaryotic promoter regions were selected by scanning with a hand-held UV illuminator (excitation: 365 nm), and further analyzed by nucleotide sequencing.

### Generation of serial deletions for the fine-mapping of core promoter region

The core promoter region of the putative promoter regions were further analyzed by directional serial deletions by using the Erase-a-Base^®^ system (Promega, Madison, WI, USA) which is based on the specific digestion of target DNA at a uniform rate with exonuclease III from a 5’-protruding or blunt-end restriction site. To create the proper DNA ends for unidirectional digestion by exonuclease III, the candidate plasmid pGP613-887 was digested at the multiple-cloning-site region of the vector upstream of the candidate promoter sequence with *Kpn*I and *Spe*I to produce a 3’-protruding end, which is resistant to exonuclease III digestion, and a 5’-protruding-end, which is susceptible to exonuclease III digestion, respectively. The products were then digested by exonuclease III to generate unidirectional serial deletions from the *Spe*I site into the 5’-terminus of the candidate promoter sequence. Aliquots of the digestion products were removed every 30 seconds and subjected to digestion with S1 nuclease to create the blunt-ends. The digestion products were then self-religated and transformed into *E. coli*, and screened for promoter activity using a hand-held UV lamp. Colonies retaining the promoter activity were selected and sequenced as described previously.

For further fine mapping of the AV3 promoter core region, pGP741-869, pGP762-869 and pGP783-869 were constructed. Forward primers AY741F, AY762F and AY783F (Table 1) were used to amplify the corresponding regions as indicated by the names of the primers of the AYVV-NT genome with the reverse primer AY869R. The PCR fragments were cloned into the pGlow-TOPO^®^ vector for the promoter activity assay.

**Table 1:** Primers used in this study.

Primer name	Purpose^1^	Oligonucleotide sequence (5’ → 3’)^2^	Position^3^
Promoter trap	A	CC A TATCTATATC**TCCT**TNNNNN	NA
AY869R	B	CC A TATCTATATC**TCCT**TGCATATTGACCC	AY869-858
AY741F	B	TATGGATTTTGGTCAGG	AY741-757
AY762F	B	TAACATGTATGATAATGAGC	AY762-781
AY783F	B	CAGTACTGCTACTATCAAG	AY783-801
C741-869F	C	GCATATTGACCCCCTG	AY869-854
C741-869R	C	CC A TATCTATATC**TCCT**TTATGGATTTTGGTC	AY741-754
nRBS831F	D	AAGGGCAATTCTGCAGATC	NA
nRBS831R	D	TAAAACTTGATAACGGTCT	AY831-813
AY889R-G	E	GCCAACGCCTGTTCCTTAG	AY889-872
AY1062R-G	E	GATTCTGAACAGAATCATA	AY1062-1046
repPF	F	AATTGCGAGCACGAATTACTG	AY291-271
repPR	F	CC A TATCTATATC**TCCT**TTTGACTTGGTCAATCGG	AY2603-2618
*rrn*B P1-F	G	CCTCTTGTCAGGCCGGAATAACTCCCTATAATGCGC	NA
*rrn*B P1-R1	G	TTATCCGCTCACAATTCCCTGGTGGCGCATTATAGG	NA
*rrn*B P1-R2	G	AACAAAATTATTTCTAGAGGGGAATTGTTATCCGCTC	NA
*rrn*B P1-R3	G	CC A TATCTATATC**TCCT**TTTAAAGTTAAACAAAATTATT	NA
TLCV762F	H	TAATATGTATGATAATG	TL762-779
TLCV869R	H	CC A TATCTATATC**TCCT**TGCATACTGACCGCCA	TL869-855
GoMV771F	H	CAACATGTACGACAATG	GO771-788
GoMV878R	H	CC A TATCTATATC**TCCT**TGCGTATTGACCTCC	GO878-865
SqLCV747F	H	TAATATGTTTGATAATG	SQ747-764
SqLCV854R	H	CC A TATCTATATC**TCCT**TGCGTATGTTCCTCC	SQ854-841

^1^ The main purpose for the primer. A, for the random amplification of fragments of the AYVV-NT genome; B, for fine mapping of AV3 promoter region by PCR; C, for molecular characterization of the AV3 promoter region; D, for verifying the function of the putative endogenous RBS; E, for verifying the translatability of the two short ORFs downstream of the AV3 promoter; F, for construction of the Rep promoter sequence of the AYVV-NT genome; G, for construction of the *E. coli rrn*B P1 promoter; H, for cloning of the corresponding region of AV3 promoter from other begomoviruses.

^2^ The bold letters indicates the position of the RBS (in the complementary sense of AGGA), and the start codon (ATG) is underlined.

^3^ The relative position of the primer sequences in the relevant viral genome. AY, AYVV-NT; TL, TLCV; GO, GoMV; SQ, SqLCV; NA, not applicable.

### Quantification of fluorescence

The promoter activity levels were quantified by measuring the fluorescence of the GFP reporter by using an FLx800™ Multi-Detection Micro-plate Reader (BioTek Instruments, Winooski, VT, USA). Individual constructs were transformed to TOP10 *E. coli* and incubated in 2 ml of LB broth containing 100 µg of ampicillin ml^-1^ at 37 °C for 16 h. Aliquots of 200 µl liquid cultures were loaded into a 96-well micro-titer plate with four duplicates, and the fluorescence was measured at an excitation wavelength of 400 nm and an emission wavelength of 508 nm at a sensitivity setting of 60. The results were analyzed using Gen5 Data Analysis Software (BioTek Instruments, Winooski, VT, USA), and the fluorescence data were normalized against the concentration of each culture at OD_600_. Fluorescence was expressed as arbitrary units (AUs). For comparing various constructs, the fluorescence of *E. coli* cells harboring the pGP613-887 plasmid was set at a value of 1000 as described [22].

### Characterization and comparison of promoter strengths

For constructs used in assays for promoter polarity, the function of downstream putative endogenous RBS, and the translatability of downstream ORFs, the construction processes were described in the Results section pertaining to each construct under study, since the purposes, designing details, and the expected consequences for these constructs were related to the relevant results of analyses on the previous mutants. The primers used for the construction of the clones by PCR or inverse PCR were listed in Table 1. The identities of these mutants were verified by nucleotide sequencing.

To amplify the Rep gene promoter of AYVV-NT as a control, the primer pair corresponding to nts 2603- 291 of the AYVV-NT genome were used (Table 1). The amplification products were cloned into the pGlow-TOPO^®^ vector, verified by sequencing, and used for comparison in the fluorescence assays. As another positive control for the comparison of endogenous constitutive promoter activity, the plasmid containing *rrn*B P1 core promoter of *E. coli* [23] was generated by performing mega-primer PCR [24] with four primers as listed in Table 1, with the initiation codon underlined and the RBS in boldface. The amplification products were cloned and analyzed as described.

### Northern blot assay

The promoter activities were also monitored by northern blot analysis of the total nucleic acids from each construct using probes specific to the GFP gene. Individual clones were cultured as described, and the total nucleic acids of each clone were extracted by using a lysis buffer (0.4 M NaCl, 40 mM EDTA, 1% β-mercaptoethanol, 1% sodium dodecyl sulfate (SDS), 20 mM Tris at pH 7.4) and hot phenol, followed by ethanol precipitation. Aliquots of the total nucleic acids, corresponding to those from 0.5 ml of culture, were analyzed by electrophoresis through a 1% agarose gel, followed by staining with ethidium bromide (EtBr), or by northern blot.

For the northern blot analysis, the nucleic acids in agarose gels were transferred to nitrocellulose (NC) paper and hybridized with digoxigenin (DIG)-labeled probes corresponding to the antisense sequence of Cycle 3 GFP, followed by detection with anti-DIG-alkaline phosphatase (AP) conjugate (Roche Diagnostics, Mannheim, Germany).

### Western blot assay

To verify the expression of GFP proteins, western blots were performed using GFP-specific antiserum. Individual clones were cultured as described, and the optical density of the culture (monitored by OD_600_ readings) was adjusted to the same value by using the LB medium. Aliquots of 16 µl liquid broths of individual clones were mixed with 4 µl 5X SDS loading dye and boiled at 100 °C for 5 min, followed by electrophoresis through a 12.5% polyacrylamide gel containing 1% SDS. The proteins in the gel were subsequently transferred to the PVDF membrane, and the presence of GFP was detected by using an anti-GFP first antibody, followed by a goat anti-rabbit IgG-AP conjugate (SIGMA-Aldrich, St. Louis, MO, USA) as the secondary antibody. The presence of GFP protein was revealed by color development using nitro-blue tetrazolium (NBT) and 5-bromo-4-chloro-3-indolyl phosphate (BCIP) (Promega, Madison, WI, USA).

### Sequence analyses

For identifying related prokaryotic promoter sequences in the bacterial subsets of GenBank [25], the highly sensitive database searching tool, the SSearch [26] program in the SDSC Biology Workbench (http://workbench.sdsc.edu) was used.

## Results

### A novel prokaryotic promoter was identified in the genome of AYVV-NT

We have shown previously that a monomeric construct of AYVV-NT is capable of producing unit-length single-stranded circular genomic DNAs and various DNA forms possibly involved in the replication of AYVV-NT in *E. coli* [19]. To explore whether unknown prokaryotic promoters exist in the genome of AYVV-NT, a promoter trapping method (Figure 1A) was adopted as described in Materials and Methods. A primer was designed to randomly amplify portions of the AYVV-NT genome for the direct cloning and analysis of promoter activities in *E. coli*, using GFP as a reporter. The expression of the GFP reporter in *E. coli* requires an active transcription regulatory element, the promoter, and an efficient translation regulatory element, the RBS, since the promoter trapping vector pGlow-TOPO is originally designed for assays in mammalian systems and dose not contain a prokaryotic RBS. In addition, the proper spacing between the RBS and the translational initiation codon (ATG) is required for efficient translation, but the GFP ORF in the pGlow-TOPO vector is 23-nt downstream to the insertion site of candidate promoter sequences. Therefore, the promoter trap primer used in this study was designed to contain an artificially added RBS, the 9-nt spacer sequence of pET21d (Novagen, Billerica, MA, USA [21]), and an initiation codon in-frame to the GFP ORF on the pGlow-TOPO vector. Consequently, if the cloned PCR fragment exhibits promoter activity and drives the transcription of mRNAs for the reporter, the translation of the mRNA would initiate from the artificially added start codon on the primer, and the resulting reporter GFP translated from these constructs is a fusion protein containing eight extra amino acids derived from the 3’-terminus G on the primer plus the 23-nt fragment downstream from the TOPO-cloning site in addition to the authentic GFP reporter ORF. In prokaryotes, transcription and translation are closely coordinated [27] since there is no nuclear membrane to separate the DNA genome and RNA transcripts from the ribosomes in the cytoplasm, thus the ribosomes can start translating the mRNA while the transcription is still in progress, as long as proper translational regulation elements such as the RBS and a properly spaced downstream ORF are exposed. To simplify the analyses, the detection of reporter GFP fluorescence was used as an indirect indicator to determine whether the promoter assayed was active for most constructs in this study. However, it should be noted that the expression of GFP reporter involves controls by both transcriptional and translational elements.

The amplification results showed that the majority of the PCR products were 200-500 bp in length. Bacterial colonies harboring the genomic fragments exhibiting prokaryotic promoter activities were revealed by using a portable UV illuminator, as exemplified in Figure 1B and 1C. More than 7500 colonies were screened, theoretically covering the whole genome of AYVV-NT, in both virion- and complementary-sense directions. The GFP-expressing clones were selected for sequence analysis, and the results revealed that all clones harbored fragments of the AYVV-NT genome spanning the region from nucleotide position nt 613 to nt 1022. The clone containing the shortest fragment, from nt 613 to nt 887 of the AYVV-NT genome (Figure 1A), was designated as pGP613-887 and subjected to further analyses. However, no previously known promoters of begomoviruses functioning in bacteria [3–5], such as those of the Rep or transcription activator protein (TrAP) genes, were identified in our surveys, probably due to their relatively lower promoter activities or the lower sensitivity of the portable UV illuminator assay and the limitations of the naked eye.

Judging from the relative direction upstream of the GFP reporter, the novel prokaryotic promoter could potentially drive the transcription of AYVV-NT genes in the virion-sense. However, the two known ORFs encoded on the virion-sense strand of AYVV-NT, the movement protein (MP) (nts 132-482) and the CP ORF(nts 292-1065), are both located upstream from the prokaryotic promoter identified in the current study, and are therefore unlikely to be under its control. No other promoter activities in this region of geminivirus genomes have been reported previously. This novel prokaryotic promoter is designated the AV3 promoter, representing the third promoter in the virion-sense of the AYVV-NT genome.

### Fine mapping of the AV3 promoter

**Figure 2:**
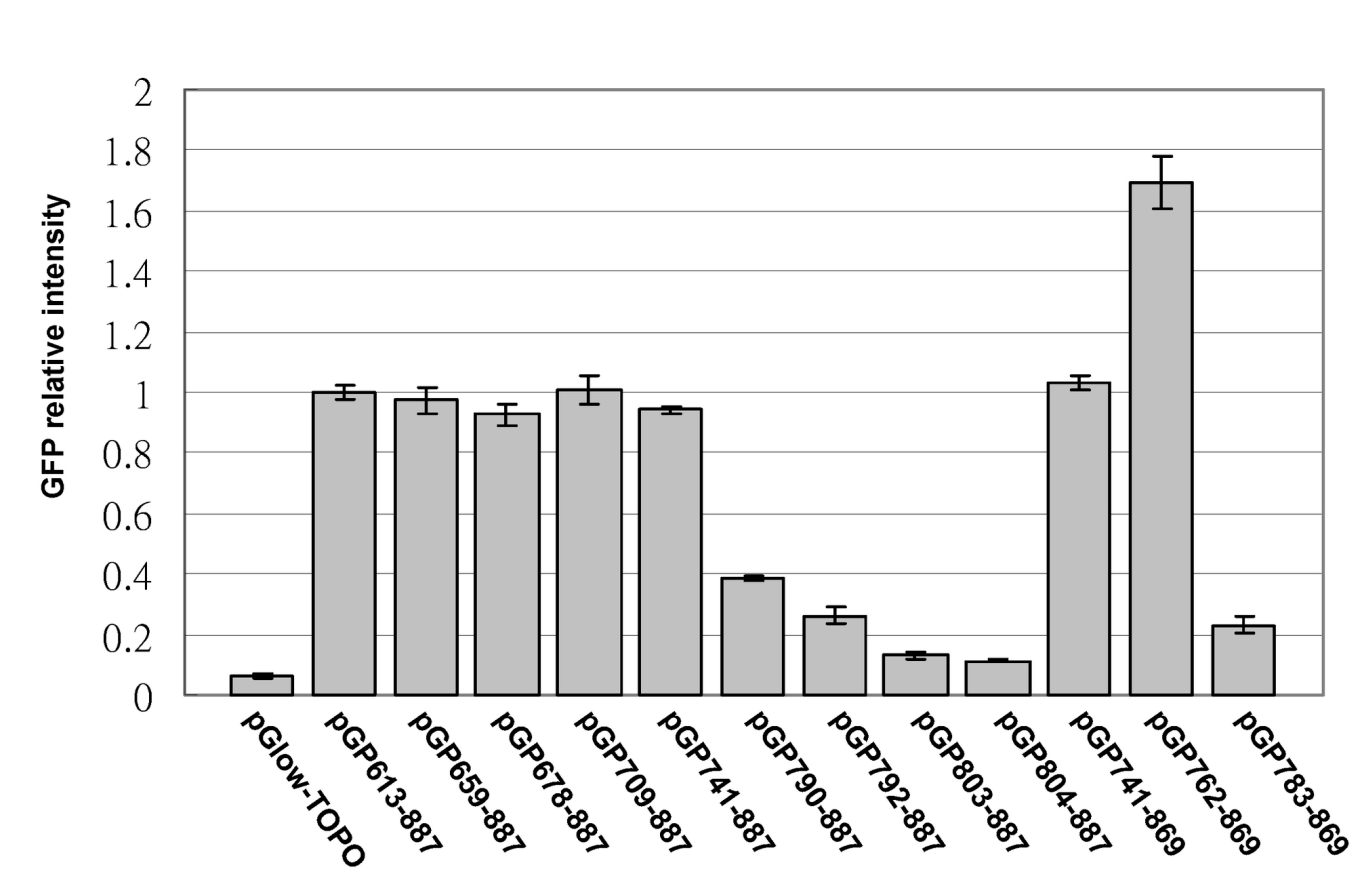
Fine mapping of the essential AV3 promoter region. Bar chart representation of the quantification of GFP fluorescence intensities of various constructs. The average fluorescence intensities of four replicates from pGP613-887 were used as the basis for comparison, with those from other constructs expressed as relative fold changes (Y axis). The identities of the constructs are indicated at the bottom of each bar. The error bars indicate standard deviations calculated from at least four replicates.

To identify the core region of the AV3 promoter, directional serial deletions were performed using pGP613-887 as the template. A series of 5’-terminus deletion clones were generated and subjected to promoter activity assays. These deletion clones are named pGPxxx-887, in which xxx indicates the 5’ nucleotide position relative to the AYVV-NT genome. Sequence analysis confirmed that the clones harbored 5’ deletions ranging from nt 613 to nt 804. The GFP expression levels of various clones were quantified using a fluorescence micro-plate reader (FLx800, BioTek) to identify the minimum-sized fragment that retained promoter activity. The results revealed that the deletion mutant pGP741-887, harboring nts 741-887 of the AYVV-NT genome, exhibited similar promoter activity to that of the original clone pGP613-887 (Figure 2), and was therefore selected for further characterizations as described in the following sections. Deletions up to the positions of nt 790 or nt 792 resulted in approximately a 60% and a 75% reduction of the promoter activity, respectively, whereas further 5’-deletions nearly abolished AV3 promoter activity (Figure 2 Table S1).

To further narrow down the core AV3 promoter region, PCR was performed with specific primers (Table 1) designed to generate progressive 20-nt 5’- or 3’-deletions on the pGP741-887 template. Results of fluorescence assays revealed that the 3’ deletion mutant pGP741-869 still retained similar promoter activity, whereas mutant pGP783-869 exhibited a severely reduced promoter activity (Figure 2). Unexpectedly, the promoter activity of pGP762-869 was 1.6 fold higher than that of pGP613-887, suggesting the existence of certain negative regulatory element in the region from nt 613 to nt 762. Based on these results, the sequences essential to and sufficient for AV3 promoter activity was determined to be located between nts 762-869 of the AYVV-NT genome.

### AV3 promoter is unidirectional

**Figure 3:**
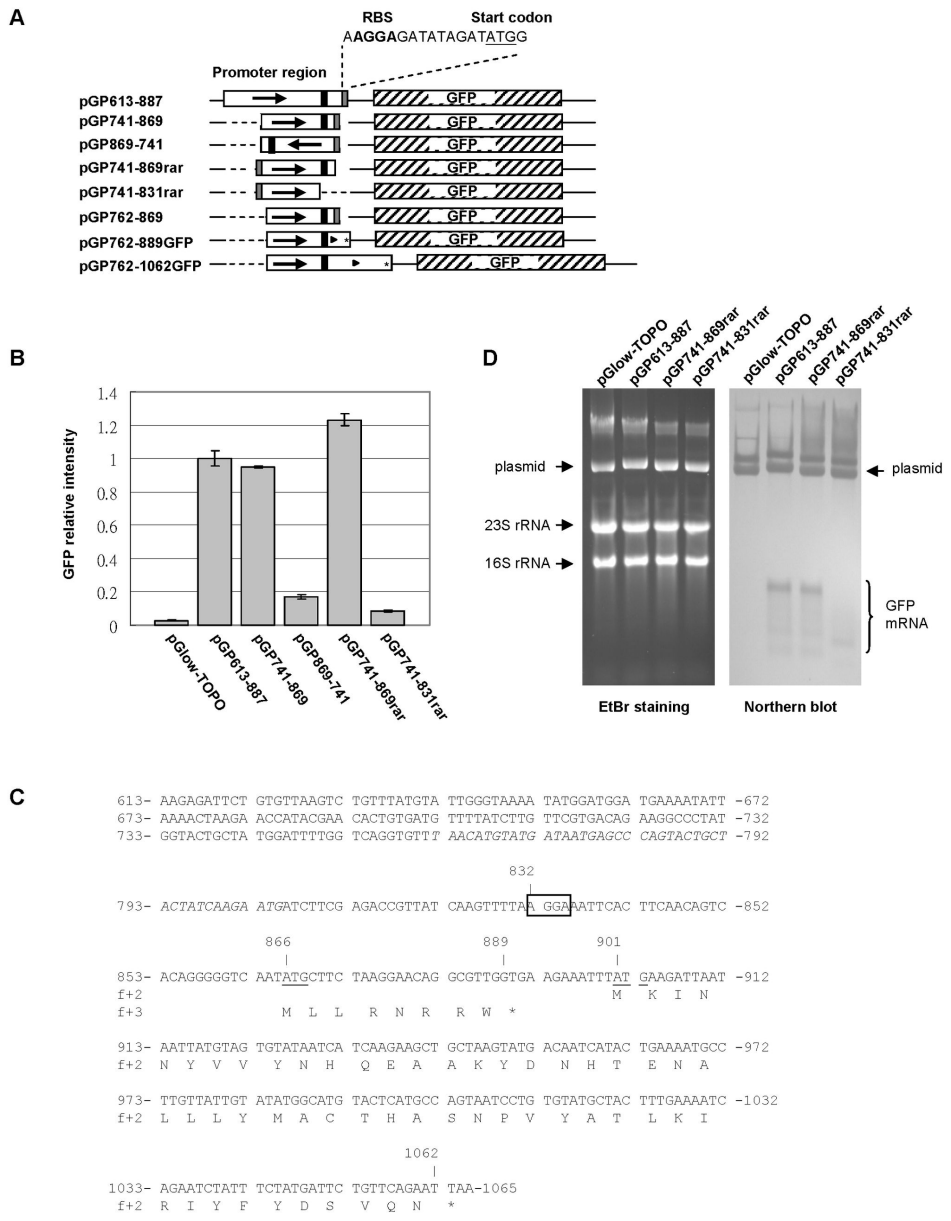
Characterization of AV3 promoter activities. (A) Schematics of the constructs used for the analyses of polarity, putative endogenous RBS and the translatability of downstream ORFs. The identities of the constructs are as indicated on the left, and the GFP reporter gene on the vector is shown as the hashed box. Promoter regions are represented by the open boxes, with the arrows heads indicating the orientation relative to the AYVV-NT virion-sense strand. The grey and black bars in the promoter regions represent the artificially added RBS and the endogenous RBS, respectively. The expected translation start codons and the mutated stop codons on pGP762-889GFP and pGP762-1062GFP are indicated by the triangles and asterisks, respectively. (B) Characterization of the AV3 promoter by fluorescence quantification as described in Figure 2. (C) Nucleotide sequence of the AYVV-NT AV3 promoter region under study, and the respective translation products of two putative downstream ORFs. The relative nucleotide positions are indicated by the numbers on the sides. The putative endogenous RBS (-AGGA-), and the start codons (ATG) for the two downstream ORFs are boxed and underlined, respectively. The two downstream ORFs, which are in different frames, with the first and the second ORF in frame +3 and +2, respectively, relative to nt 1 of the AYVV-NT genome, as denoted by f+3 and f+2. The amino acid sequences of the translation products of each ORF are shown in one-letter code underneath, with an asterisk indicating the stop codon. The region containing the putative -35 and -10 boxes that was used for sequence analyses (Table S2) is italicized. (D) Analysis of GFP reporter mRNA accumulation by northern blot hybridization. Total nucleic acids from *E. coli* harboring different constructs (as indicated at the top) were separated by electrophoresis through a 1% agarose gel, and stained by EtBr or transferred to a NC membrane followed by hybridization with DIG-labeled probes specific to GFP mRNA. The positions of the ribosomal RNAs (rRNA), the plasmid DNAs, and GFP mRNAs are indicated.

Geminiviruses use a genome strategy involving bi-directional transcription from a double-stranded DNA intermediate [28–31]. Analysis of AYVV-NT sequence revealed the presence of a potential complementary-sense ORF (nts 687-562) near the AV3 promoter. To verify whether the AV3 promoter could drive the transcription in the complementary-sense direction, we constructed pGP869-741 with the primer pair, C741-869F and C741-869R (Table 1), to amplify and clone the nts 741-869 region of AYVV-NT in the inversed direction (black arrow, Figure 3A) relative to the artificially added RBS, 9-nt spacer, and a start codon on primer C741-869R (Figure 3A). Since the PCR product could be cloned into the pGlow-TOPO in both directions, two constructs, pGP869-741 and pGP741-869rar, containing the same PCR fragment in opposite directions relative to the GFP ORF on the vector were obtained (Figure 3A). The “rar” in the name stands for “reversed artificial RBS” to indicate that the artificially added RBS is in the reversed direction relative to the GFP ORF on the vector in pGP741-869rar, which was not supposed to drive the expression of GFP. Both constructs were subjected to promoter activity assays by using fluorescence detection. The results revealed that GFP fluorescence was severely reduced for pGP869-741 (Figure 3B) with the AV3 promoter region reversed, even though the elements required for translation (the artificially added RBS/spacer/start codon, grey box in Figure 3A) were in the correct direction relative to the GFP ORF on the vector (Figure 3A), suggesting that the promoter activity was severely impaired. This result indicated that the AV3 promoter region is unidirectional.

Unexpectedly, pGP741-869rar exhibited similar or slightly higher GFP fluorescence level as compared to that of pGP741-869 (Figure 3B) although the artificially added elements for translation (RBS/spacer/start codon) were in the reversed direction relative to the GFP ORF on the vector. The result suggested that there might be a functional endogenous RBS and a translatable start codon downstream from the AV3 promoter region in nts 741-869 of the AYVV-NT genome. Sequence analysis revealed the presence of a putative RBS, (-AGGA-), at nts 832-835 of the AYVV-NT genome (hereafter referred to as the putative endogenous RBS, represented by the black bars and boxed region in Figure 3A and 3C, respectively), in addition to two plausible downstream ATG translational start codons (Figure 3C, underlined). Although not essential for transcription, the RBS and a properly spaced downstream start codon are required for the translation of the mRNA transcripts in *E. coli*. To test the function of the putative endogenous translational control elements, pGP741-831rar was constructed (Figure 3A) by using inverse PCR with the primer pair nRBS831F and nRBS831R (Table 1) to delete the putative endogenous RBS (nts 832-835), the 30-nt downstream spacer (nts 836-865) plus the first start codon (nts 866-868), from the template pGP741-869rar. The expected GFP mRNA transcript from pGP741-831rar should contain the region from the transcription initiation site to nt 831, plus a 23-nt vector region downstream from TOPO-cloning site to the GFP ORF of the pGlow-TOPO vector, plus the whole GFP ORF and the 3’-untranslated region. If there are other endogenous translational control elements upstream from nt 831, the GFP reporter should be translated to the level comparable to that of pGP741-869rar. However, the results of GFP fluorescence assays showed that the GFP expression level of pGP741-831rar was severely reduced, suggesting that the putative endogenous RBS should be located downstream from nt 831, and the spacer and at least the first start codon are essential for the expression of the reporter gene.

To distinguish the effects on transcription and on translation, northern blot analysis was performed. As shown in Figure 3D, both pGP741-869rar and pGP741-831rar retained the promoter activity for the transcription of the mRNA for GFP, as did the positive control pGP613-887. Although the GFP mRNAs isolated from *E. coli* harboring pGP741-831rar appeared smeared, the signals suggested that the promoter region was functional. The instability of the GFP mRNAs might be attributed to the loss of translation on these mRNAs due to the lack of the putative endogenous RBS, or could be resulted from the inability of the truncated promoter region (nts 741-831) to initiate transcription from a precise position. The translatability of the mRNA and other factors that influence the loading of ribosome onto the mRNA are important determinants of mRNA stability [32,33]. If the smeared GFP mRNA signals were the transcripts initiated from imprecise sites due to the deletion, some of the imprecisely initiated transcripts might contain a functional RBS. Then the stability of those transcripts with a functional RBS would increase with the loading of ribosomes and could potentially be detected at a similar position as those seen for pGP613-887 and pGP741-869rar in the northern blot (Figure 3D). However, no visible signals were detected for pGP741-831rar near the GFP mRNA positions of pGP613-887 and pGP741-869rar (Figure 3D). Thus, the smeared mRNA signals at the lower part of the blot observed for pGP741-831rar suggested that the endogenous RBS likely was truncated in the mutant, although the possibility of the imprecise transcription initiation events could not be ruled out and the location of the actual endogenous RBS remains to be verified by mutational studies. Collectively, these results demonstrated that the AV3 promoter is unidirectional and suggested that AYVV-NT genome likely harbors an endogenous prokaryotic RBS downstream from the AV3 promoter region.

### Downstream ORF of the AV3 promoter

**Figure 4:**
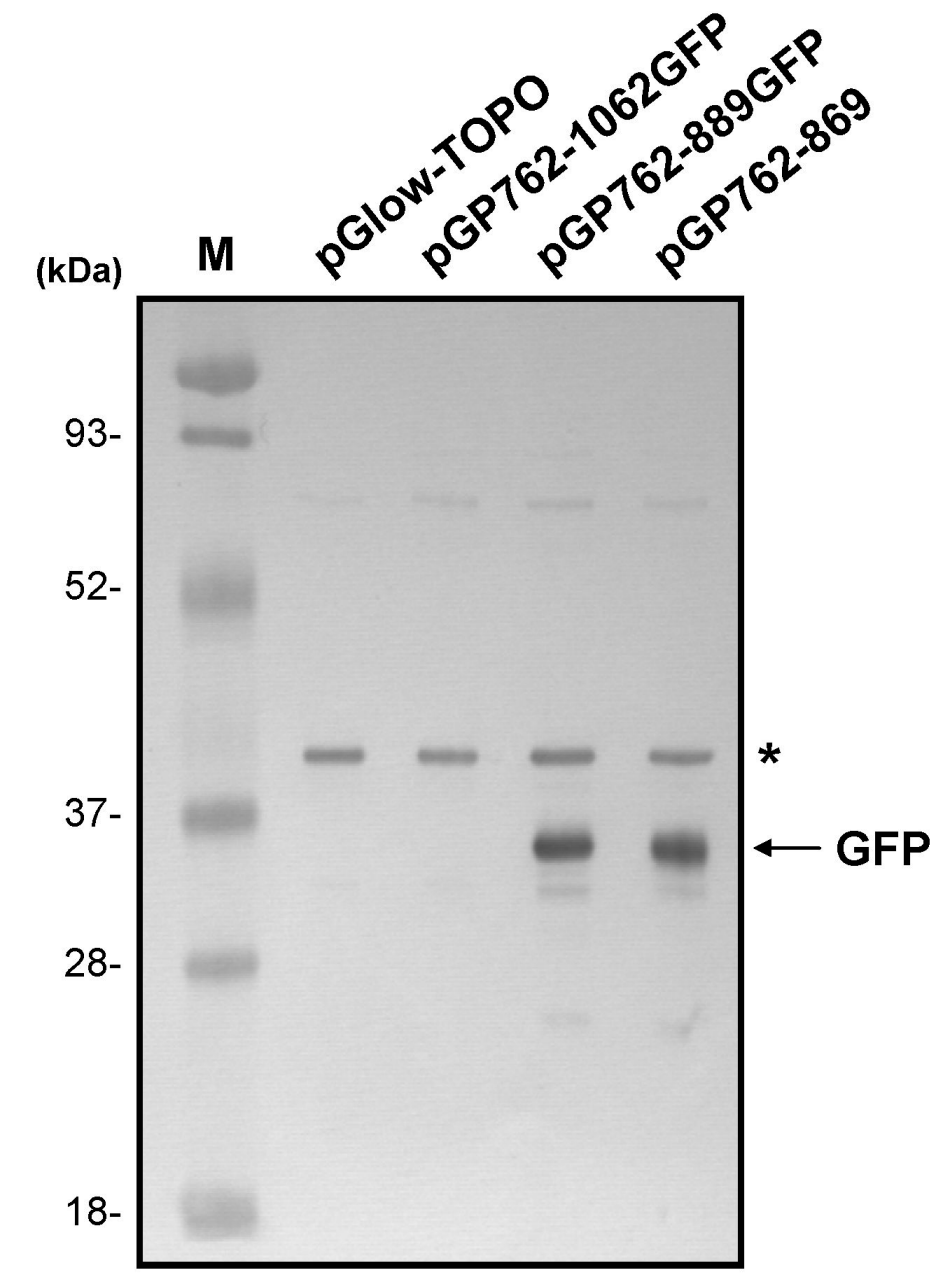
Translation of the putative downstream ORFs as detected by western blot analysis. Total proteins from *E. coli* harboring various constructs (as indicated at the top) were separated by electrophoresis through a 12.5% polyacrylamide gel containing 1% SDS, transferred to a PVDF membrane, and probed with a GFP-specific antiserum. The relative molecular weight of the size marker (lane M) is shown on the left. The position of GFP is indicated on the right. A non-specific protein band is indicated by an asterisk. The expected molecular weight of the GFP-fusion protein expressed from pGP762-889GFP and the positive control from pGP762-869 are 28.96 kDa and 28.62 kDa respectively, as calculated from the amino acid sequence.

Computer-aided sequence analysis revealed the presence of two short ORFs, initiating from the two downstream ATG codons, nts 866-892 and nts 901-1065, downstream from the AV3 promoter region (Figure 3C). The two short ORFs are in different frames, with the first and the second ORF in frame +3 and +2 relative to nt 1 of the AYVV-NT genome, as denoted by f+3 and f+2 in Figure 3C, respectively. It should be noted that the second ORF (nts 901-1065) overlaps with the C-terminus of the CP ORF (nts 292-1065) for AYVV-NT. To further verify the translatability of the two short ORFs, western blot analyses were performed using two GFP-fusion constructs, pGP762-889GFP and pGP762-1062GFP. For the construction of pGP762-889GFP, nts 762-889 of AYVV-NT genome, spanning the core AV3 promoter region, the putative endogenous RBS, the spacer, and the first ORF, were amplified using the primer pair AY762F and AY889R-G with pAYVV-NT as the template. The fragment spanning nts 762-889 of AYVV-NT genome, plus an extra C to mutate the stop codon (as indicated by the asterisk in Figure 3A), was amplified and cloned into the TOPO-cloning site of the pGlow-TOPO vector as described previously. The original stop codon (UGA) at nts 890-892 was replaced by the C to generate a fusion reporter ORF containing the first short ORF (nts 866-889), a C, and a 23-nt vector region downstream to the TOPO-cloning site and the reporter GFP ORF of the vector. Similarly, pGP762-1062GFP, was constructed by PCR amplification of nts 762-1062 of the AYVV-NT genome, plus an extra C, by primer pair AY762F and AY1062R-G. The original stop codon (UAA) at nts 1063-1065 was replaced by the C to generate a fusion reporter ORF containing the second short ORF (nts 901-1062), a C, and a 23-nt vector region downstream to the TOPO-cloning site, and the reporter GFP ORF. Note that the transcription initiation site downstream from AV3 promoter region and the putative RBS of the two constructs are the same, but the two ORFs are in different frames, with different lengths of spacer. The purpose of design is to test the translatability of the two short ORFs on mRNA transcripts initiated from the same AV3 promoter region. The total protein extracts from *E. coli* harboring pGP762-889GFP or pGP762-1062GFP were subjected to western blot analysis by using a GFP-specific antiserum, with those from *E. coli* harboring pGlow-TOPO or pGP762-869 as negative and positive control, respectively. The result revealed that the first ORF in frame +3, encompassing nts 866-889, was translatable (Figure 4) in contrast to the second downstream ORF.

### AV3 promoter is a strong and constitutive prokaryotic promoter

**Figure 5:**
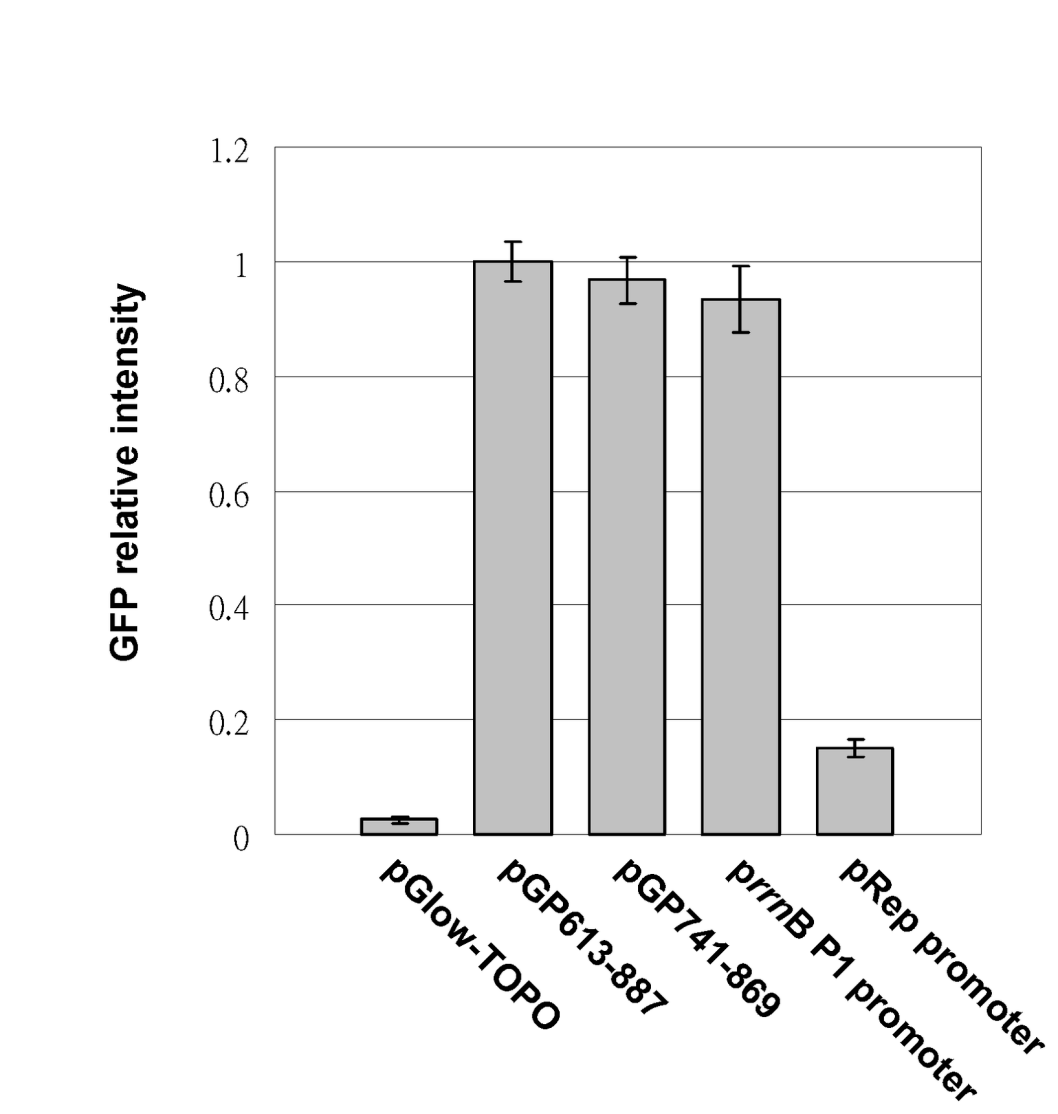
Comparison of promoter strengths. The strengths of different AV3 promoter regions (as indicated) were compared to that of the constitutive promoter, *rrn*B P1, and to the promoter for the Rep gene by fluorescence quantification as described in Figure 2.

Previous studies have shown that certain geminivirus promoters are functional in prokaryotic systems [3–5,34]. Among these, the promoter corresponding to the Rep gene has exhibited the highest activity in bacteria [3,4]. To assess the strength of the AV3 promoter, the corresponding promoter region for the Rep gene of AYVV-NT, nts 2603-291, was cloned into the pGlow-TOPO^®^ vector and used for comparison in promoter activity assays. In addition, since these geminivirus promoters appeared to be constitutively active without requiring induction, a strong constitutive promoter of *E. coli*, *rrn*B P1 [23], was also cloned into the pGlow-TOPO^®^ vector and assayed as the basis for comparison. Results of the fluorescence assays (Figure 5) revealed that the promoter activities of pGP613-887 and pGP741-869 were nearly ten times higher than that of the Rep promoter (Figure 5 Table S1). In addition, the AV3 promoter exhibited similar or slightly higher activities compared to the well-studied constitutive *rrn*B P1 promoter (Figure 5 Table S1).

### Sequence analyses of the AYVV-NT AV3 promoter

**Figure 6:**
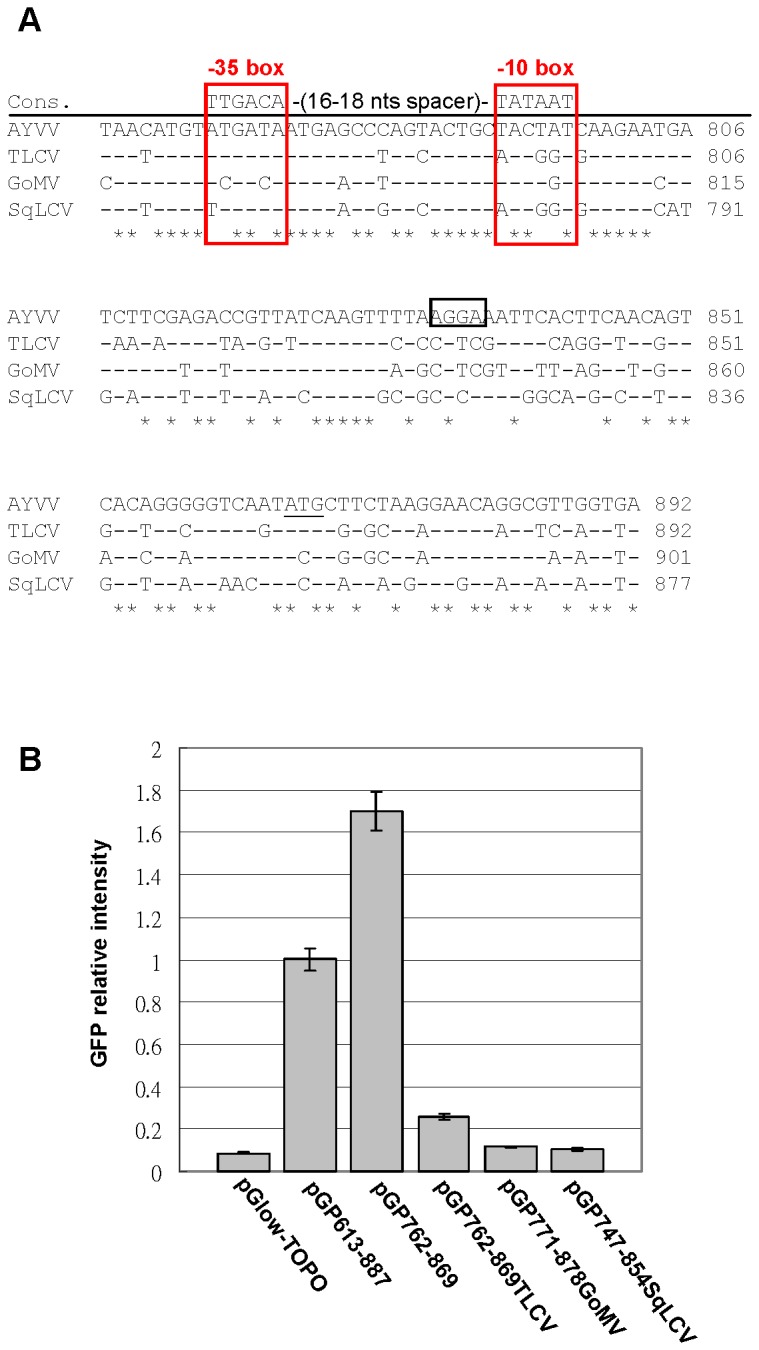
Analysis of promoter activities in the regions corresponding to the AYVV-NT AV3 promoter in various begomoviruses. (A) Comparison of nucleotide sequence similarities among the AV3 promoter corresponding regions in different begomoviruses. The respective nucleotide positions are indicated by numbers on the right. The consensus sequence of prokaryotic promoter containing the -35, -10 regions (in red boxes) are shown above the alignment for comparison. Nucleotide sequences identical to that of the AYVV-NT are represented by a dash. Conserved sequences among all sequences are indicated by an asterisk under the alignment. The putative endogenous RBS (-AGGA-), and the start codon (ATG) of the AYVV-NT sequence are in black box and underlined, respectively. (B) Relative promoter activities of the AV3 promoter corresponding regions from various begomoviruses were quantified as described in Figure 2.

The discovery of a novel prokaryotic promoter in the AYVV-NT genome prompted us to search for the presence of similar promoters in the genomes of other begomoviruses. The AV3 promoter corresponding regions (Figure 6A) from several begomovirus isolates collected by our laboratories, including TLCV [19], SqLCV [20] and GoMV [19], were cloned and assayed for promoter activity. All three of the begomoviruses exhibited low levels of AV3 promoter activity compared to that of AYVV-NT (Figure 6B). Their corresponding AV3 promoter regions of all four begomoviruses harbored sequence elements similar to those of consensus -10 and -35 boxes [35,36], with a 16-nt spacer (Figure 6A). However, the sequences were less conserved in the -10 box, with one substitution for GoMV, and three substitutions for TLCV and SqLCV compared to that of AYVV-NT. Higher nucleotide sequence similarity between the corresponding AV3 promoter regions of AYVV-NT and TLCV seemed to contribute to the relatively higher promoter activity of TLCV.

Evidence suggests that geminiviruses might have originated from the plasmids of prokaryotes or primitive eukaryotic hosts [14,16,18,37,38]. The presence of the AV3 promoter in the AYVV-NT and other begomovirus genomes provided further evidence to support this notion.

To explore further the possible prokaryotic origins of the AV3 promoter sequences, a detailed search of the known *E. coli* promoter database, RegulonDB [39], and the bacteria subsets of GenBank [25], were performed using a more sensitive Smith-Waterman search algorithm optimized for the identification of local similarities [40]. The AYVV-NT AV3 promoter was found to share the highest sequence similarities to *E. coli* promoters under the regulation of Sigma70 or Sigma24 transcription factors in more than 3000 candidate alignments, with a 56.6% similarity to the promoter ECK125137608 regulated by Sigma70. In the candidate hits in the bacterial subset of GenBank with nucleotide similarity of over 70% and an E-value lower than 0.4, the AYVV-NT AV3 promoter sequence was found to resemble the known promoter sequences present in six bacteria species (Table S2). These bacteria were found either in the digestive systems ( *Lacococcus* *garvieae* [41]) or in some extreme conditions in marine or fresh-water environments [42–46]. The result provided further evidence to support the notion that geminiviruses might have a prokaryotic evolutionary origin from bacteria that reside in a more primitive environment.

## Discussion

In this study, we identified a novel prokaryotic promoter, the AV3 promoter, encompassing nts 762-869 of the AYVV-NT genome near the 3’-terminus of CP ORF. No known geminivirus genes have been reported previously in proximity downstream of the AV3 promoter. The discovery of such a prokaryotic promoter provided further evidence in support of a prokaryotic origin in the evolutionary history of geminiviruses. The strong and constitutive activity of the AV3 promoter also could have biotechnology applications.

The AV3 promoter is unidirectional in the virion-sense (Figure 3B) of the AYVV-NT genome, with strength comparable to that of the strong endogenous *rrn*B P1 promoter of *E. coli*. A short ORF encoding a peptide of eight amino acids was also found to be translated in *E. coli*, regulated by the AV3 promoter and an endogenous RBS (Figure 4). The potential biological function of the short ORF is currently unknown and requires further mutational and functional studies, but the presence and translatability in *E. coli* of the short ORF suggested that AV3 promoter region is a remnant of the prokaryotic origin and not just nucleotide sequences coincidently exhibiting prokaryotic promoter activity. The putative endogenous RBS at nts 832-835 shares similarity with other canonical Shine-Dalgarno sequences [47–49], especially for the 4-nucleotide motif, -AGGA- [49] (Figure 3C).

In addition, promoter activity assays of the serial deletion mutants revealed the existence of a putative negative regulatory element at nts 613-761 in the AYVV-NT genome. Deletion of nts 613-761 resulted in an approximately 1.6 fold increase in the promoter activity. Negative regulation is a common regulatory mechanism of prokaryotic operons, in which the operator sequences are located in proximity to or even overlap with the promoter sequences for proper control by the repressors, which could be de-repressed in the presence of inducers [50,51]. Although the AV3 promoter appeared to be a constitutive promoter without requiring induction, it is possible that nts 613-761 of AYVV-NT genome could serve as a negative regulatory element that interacted with certain repressor proteins in *E. coli*. We found the AV3 promoter sequence in some other monopartite begomoviruses (TLCV and GoMV), but the activity of the promoter sequences of these viruses was considerably less than that of AYVV-NT (Figure 6B). none the less, the presence of these functional regulatory elements in the genomes of AYVV-NT, TLCV, and GoMV provides further supports for the prokaryotic evolutionary origins of geminiviruses.

There are two potential environments in which begomoviruses could be in proximity to prokaryotic transcription machineries in the infection cycles: in the chloroplasts [5–7] which have been shown to possess core subunits of a cyanobacterial-type RNA polymerase [9,52], and in the digestive system of whiteflies where symbiotic bacteria resides [53–57]. It has also been shown that *Agrobacterium tumefaciens* and *E. coli* support DNA replication of certain begomoviruses [3,4]. It is possible that certain begomoviruses might retain the ability to express some of their genes inside such prokaryotic systems during their infection cycles. Further mutational studies are needed to verify the potential biological function of the AV3 promoter in the begomovirus infection process.

Geminiviruses have been implicated in the first recorded plant viral disease reported in relevant literature [58,59]. The evolutionary history of geminiviruses has long been an active subject [5,11–14,16,60–62]. Although it is generally believed that geminiviruses may have originated from ancient extra-chromosomal DNAs with a rolling-circle-replication mechanism associated with prokaryotes or certain primitive eukaryotes (such as red algae [13]) [14], there are still discrepancies as to whether geminiviruses evolved from a type II phytoplasmal plasmid by acquiring a CP gene from a plant RNA virus [11] or evolved earlier from the single-stranded DNA plasmid in red algae existing 450 million years ago [13] by obtaining the CP gene from marine DNA viruses [61]. Based on the similarities between the Rep gene of geminiviruses and the plasmids of Gram positive bacteria in the pLS1 family, Koonin and Ilyina [16] proposed that geminiviruses may have evolved from bacterial plasmids. Krupovic and colleagues [11] extended this hypothesis by proposing that phytoplasmal plasmids could be the origin of geminiviruses, which might have acquired the CP gene from a plant RNA virus co-infecting the same host, based on the similarities between the Rep proteins of geminiviruses and phytoplasmal plasmids and homology-modeling analyses on CP structures. In contrast, Saccardo and colleagues [61] recently presented evidence suggesting that the type II phytoplasmal plasmid may have acquired the Rep gene from ancient geminiviruses. By analyzing metagenomic data derived from marine environments, Saccardo and colleagues identified several sequences of a possible viral origin that shared significant similarities with geminivirus CP genes, and proposed that geminiviruses may have evolved their CPs from marine environments [61]. In this study, detailed database searches revealed that the AV3 promoter shared significant similarity (greater than 70% with an E-value less than 0.4) with the promoter regions of several bacteria of marine or freshwater environment (Table S2). Thus, our findings provide further evidence in support of the notion that geminiviruses may have an ancient prokaryotic origin from marine [61] or freshwater environments.

In conclusion, we identified and characterized a novel prokaryotic promoter with strong and constitutive activity in the genome of AYVV-NT. The high strength of the AV3 promoter, the presence of a downstream prokaryotic RBS and a translatable ORF with proper spacing, and the existence of a putative negative regulatory element suggested that the AV3 promoter more likely is derived from a prokaryotic ancestor, rather than an unspecific sequence region exhibiting prokaryotic promoter activity by chance.

## Supporting Information

Table S1 **Table S1.**
(DOC)

Table S2 **Table S2.**
(DOC)
