# Supplementary material for: A Novel Prokaryotic Promoter Identified in the Genome of Some Monopartite Begomoviruses
Source: PLoS One. 2013 Jul 25;8(7):e70037. doi: 10.1371/journal.pone.0070037 (PMC3723831; doi:10.1371/journal.pone.0070037)
Supplement: Table S1 — (DOC) [file pone.0070037.s001.doc]

Table S1. Summary of the promoter activity assays by quantification of the fluorescence of GFP reporter.

| plasmid | Length of promoter region (bps) | Fluorescence (AU)^1^ |
| --- | --- | --- |
| pGlow-TOPO | 0 | 62 ± 5 |
| pGP613-887 | 275 | 1000 |
| pGP659-887 | 229 | 974 ± 43 |
| pGP668-887 | 210 | 925 ± 35 |
| pGP709-887 | 179 | 1008 ± 45 |
| pGP741-887 | 147 | 942 ± 14 |
| pGP794-887 | 98 | 385 ± 9 |
| pGP792-887 | 96 | 261 ± 28 |
| pGP803-887 | 85 | 131 ± 9 |
| pGP804-887 | 84 | 112 ± 6 |
| pGP741-869 | 129 | 1031 ± 21 |
| pGP762-869 | 108 | 1692 ± 88 |
| pGP783-869 | 87 | 231 ± 28 |
| pGP869-741 | 129 | 172 ± 12 |
| pGP741-869rar | 129 | 1231 ± 36 |
| pGP741-831rar | 91 | 85 ± 7 |
| p*rrn*B P1 promoter | 42 | 934 ± 59 |
| pRep promoter | 442 | 151 ± 16 |
| pGP762-869TLCV | 108 | 257 ± 13 |
| pGP771-878GoMV | 108 | 115 ± 6 |
| pGP747-854SqLCV | 108 | 104 ± 4 |

^1^. AU as defined by the fluorescence microplate reader (Biotech FLx800™), with a sensitivity setting of 60. The fluorescence of pGP613-887 was set to 1000, and used as the standard for comparison in all experiments after normalization to the concentration of the bacterial culture by measuring the optical density at 600 nm (OD_600_). The average readings from at least four replicates for each construct were presented followed by the standard deviations.
