# Supplementary material for: A Novel Prokaryotic Promoter Identified in the Genome of Some Monopartite Begomoviruses
Source: PLoS One. 2013 Jul 25;8(7):e70037. doi: 10.1371/journal.pone.0070037 (PMC3723831; doi:10.1371/journal.pone.0070037)
Supplement: Table S2 — (DOC) [file pone.0070037.s002.doc]

Table S2. Promoter regions from various bacterial genomes sharing high nucleotide sequence similarities with AYVV-NT AV3 promoter^1^.

| species | Accession number | Matched region^2^ | Sequence identity | E-value | Length between promoter and ORF^3^ | Possible downstream gene | Habitat | Reference |
| --- | --- | --- | --- | --- | --- | --- | --- | --- |
| Lactococcus garvieae ATCC 49156 | AP009332 | 44-2:  1578938-1578980 | 77.3% | 0.051 | 75 | polyribonucleotide nucleotidyltransferase | Pond, fish farm | [41] |
| Vibrio vulnificus YJ016 | BA000037 | 11-44:  3254573-3254606 | 76.5% | 0.17 | 168 | conserved hypothetical protein | Marine |  |
| Colwellia psychrerythraea 34H | CP000083 | 41-1:  888367-888407 | 73.2% | 0.092 | 78 | hypothetical protein | Marine |  |
| Halanaerobium hydrogeniformans | CP002304 | 36-5:  1423168-1423199 | 78.1% | 0.23 | 104 | DNA gyrase, B subunit | Lake |  |
| Nitratifractor salsuginis DSM 16511 | CP002452 | 44-13:  1633344-1633375 | 78.1% | 0.34 | 74 | response regulator receiver modulated diguanylate phosphodiesterase | Marine |  |
| Haliscomenobacter hydrossis DSM 1100 | CP002691 | 44-3:  6030454-6030493 | 73.8% | 0.16 | 227 | UvrD/REP helicase | Lake, ditch |  |

^1^. The -35 and -10 box-containing region of AYVV-NT (italicized in Figure 3C) was used as the query to search against the bacterial subset of GenBank.

^2^. The regions showing high similarities between the AYVV-NT AV3 promoter region and the genomic sequences of the respective bacteria species, respectively. The numbers are shown relative to the orientations of the genomes deposited in the GenBank. For example, “44-2” of AYVV-NT AV3 promoter refers to the complementary sense of the region on the AYVV-NT genome, whereas “1578938-1578980” represent the genomic sense of *L. garvieae*.

^3^. The lengths refer to the spacing, in nucleotides, between the end of the promoter sequence and the translation start site of the downstream ORF.
